# Lost in translation: the history of the Ebers Papyrus and Dr. Carl H. von Klein

**DOI:** 10.5195/jmla.2023.1755

**Published:** 2023-10-02

**Authors:** Jane A. Hartsock, Colin M. E. Halverson

**Affiliations:** 1 jhartsock@iuhealth.org, Indiana University Health & Indiana University School of Liberal Arts, Indianapolis, IN.; 2 chalver@iu.edu, Center for Bioethics, Indiana University School of Medicine, Indianapolis, IN.

**Keywords:** Ancient Egyptian medicine, Ebers Papyrus, Carl H. von Klein, translation

## Abstract

While the Ebers Papyrus is understood to be one of the oldest and most complete contemporaneous perspectives on Ancient Egyptian healing practices, nothing has yet been said about the biography of its first English-language translator, Dr. Carl H. von Klein. A German immigrant and surgeon in the American Midwest, von Klein spent twenty-some years meticulously translating and annotating the Papyrus, but ultimately his manuscript was destroyed. In this paper, we examine the societal- and personal-scale forces that thwarted his efforts to transform our understanding of the history of medicine.

## INTRODUCTION

“Noble Savant Ill in Charity Ward: Dr. Carl Von Klein, Scholar and Writer Who Spent Fortune in Research, Now Is Penniless.” Such is the headline of the December 8, 1913, announcement appearing in Chicago's *Inter Ocean* newspaper, advising of the hospitalization of the now-forgotten German physician. The briefly recited biography of von Klein as set forth in the article suggests notoriety: “a trustee of the John Crerar Library, a noted Egyptian [sic] scholar, author of several books and a man who sacrificed his fortune in the advancement of science.” Von Klein is further identified as a member of a “titled German family and a graduate of Heidelberg university.” The announcement appears to have been a plea to the public by the physicians caring for Dr. von Klein, that so genteel a man should not be forced to spend his final days in a facility for the impoverished, “seemingly forgotten by the friends of his prosperity” [1]. Despite their appeal, von Klein ultimately died four days later, on December 12, 1913, leaving his wife Cornelia “Nellie” von Klein a comparably young widow without a source of income.

In the more than one hundred years since his death, little of substance has been written about von Klein's contributions “in the advancement of science,” significant though they were. Much like the Egyptian text he set about translating, von Klein's personal story and works have lain forgotten for generations. Profoundly mortal, vulnerable to suffering, disappointment, and bad judgment, this obscure yet forceful polymath has disappeared from our distributed consciousness, his project forgotten even by his own family.

## CHARTING VON KLEIN'S BIOGRAPHY

Research into von Klein reveals an elusive character whose biography is complicated by several factors. His name – the noble-sounding “von” – may have been adopted after his immigration to the United States, as we have uncovered no record of any von Kleins originating in the areas of Prussia from which he claimed to have emigrated. Von Klein also, at turns, used anglicized and non-anglicized forms of his name, including Karl and Charles, and gave his middle name as Henry and Heinrich in both his publications and government-issued documents [2]. He even occasionally published under a Latinized name, Carolo H. vonKlein [3].

Von Klein was reported variously to have studied at Heidelberg [4], “the University of Prussia, at Koenigsberg,” Berlin, and Prague [5]. It is also possible that he obtained his medical training in the army, though he never asserted this. Nevertheless, many sources suggest that von Klein spent his twenties involved in the employ of several European militaries: serving as a surgeon in the Prussian Army Corps, in charge of a field hospital outside Hanover during the Franco-Prussian War of 1870, serving in the recently established Red Cross during the Serbian–Turkish Wars of 1876-1878, and finally assuming the role of surgeon in the Russian army until around 1880 [5]. It is critical to note that the absence of official government records may be the result of the bombing of Germany during World War II. Inquests at numerous archives have returned the same response: those documents have been lost. Thus, while confused and convoluted, these ranging reports do not necessarily suggest inaccuracies.

It is understandable why von Klein would have sought to affiliate himself with the formalized medical education of Prussia. To the extent he had completed his training at any of these institutions – Heidelberg, Berlin, Koenigsberg, or Prague – such a diploma would have placed him at the forefront of his field at the time. Medical education in the United States is generally held to have “professionalized” during the early twentieth century, with the publication of the so-called “Flexner Report,” which stood as the programmatic declaration of that movement.

When von Klein arrived in the United States in approximately 1870, medical education, particularly in the Midwest, was in total disarray. Illinois had thirty-nine medical schools; Indiana had twenty-seven; Cincinnati, Ohio alone had about twenty [6]. Teaching was inconsistent and predominantly didactic, with little emphasis on anatomy instruction. Entrance criteria were likewise lax and, as Flexner described, instruction was apt to take place in any location in which “a hall could be cheaply rented and rude benches were inexpensive” [6]. Even by 1910, Flexner noted that it was “as easy to establish a medical school [in the United States] as a business college” [6].

Therefore, it is important that any formal medical education would have made this German-born, American-residing surgeon part of the educated elite. Further, though we have been unable to confirm definitively his completion of such a course of study, von Klein presented himself in this manner, and it seems he was accepted by his Midwestern colleagues as a distinguished expert in part for that reason. In fact, von Klein himself applauded the “gradual evolution [toward] higher medical education” that he was witnessing in the United States, perceiving it as critical to reaching the standards that were then being set by European countries [7]. While he quit his medical practice before licensure was required in the states in which he resided, we can confirm his work through numerous newspaper accounts of his feats of healing [e.g., 8], contemporaneous lists of local physicians [9], and city directories containing his offices [10], among others.

Researching the contributions of an individual such as von Klein presents significant challenges. Our efforts to discern fact from fiction when it comes to this historical figure have spanned from the repositories of massive commercial databases such as ancestry.com to the government archives of small municipalities of the present-day German towns and villages in which von Klein is said to have resided. Ship manifests, passport applications, baptismal records, and newspaper articles concerning the personal and professional endeavors of the subject of this paper serve as the basis (often conflicting and contradictory) of our attempts to detail his life.

Through these investigations, however, a clear image of von Klein's professional and personal history in the United States emerges. In approximately 1876, he married Nellie née Zacharias, and together they reared five children. Over the first decade spent in the Midwest, he occupied himself tending to patients and traveling to scientific conferences across the country. Yet, in the midst of all those activities, he also published rapaciously on medical matters, in both local and national journals. Moreover, he was integral in founding a number of societies and taking part in the explicit professionalization of medicine in America, something that he had already witnessed transpire in his childhood home in Prussia. Specifically, von Klein was an early or founding member of the Microscopical Association of Dayton [11], the Dayton Cremation Company [12], the American Rhinological Association (ARA) [13], and the Newberry Library in Chicago [14]. He also was an editor of the popular journal *Hygiene, Diet, and Long Life* and of *JAMA* [15, 16]. In a similar vein, he proposed a deluge of new medical inventions, some of which he patented, including a gynecological stirrup, an intubation thimble, and nasal-bone forceps, as well as more quotidian objects, such as a folding cap and a “malt juice” [17-21].

Amid all this activity, von Klein nevertheless found time to continue his intellectual pursuit of languages. This interest was instilled in his childhood when he learned French, German, and Polish from his governess in Prussia [5]. In 1884, he announced that he was “preparing a medical lexicon in forty-two languages” [22]. The local penny press joked good-naturedly with the man to whom they later referred as a “regular essayist” [23]: “E pluribus unum! Zwei lager! Oui, monsieur! Erin go bragh!” [22]. Von Klein famously gave a toast “in fifteen different languages” at the annual meeting of the ARA in 1887 [24]. His Dayton home was said to house a library of thousands of texts written in French, German, Russian, and English, and upon moving to Chicago, he opened a “bureau of medical literature” which was intended to furnish texts “from every language in which medicine is written” [25]. It is perhaps unsurprising that the president of the International Medical Congress said that von Klein was of great value to his organization, “speaking as he does, nearly every language” [13].

## HIERATIC TO ENGLISH

Despite these numerous professional endeavors, if von Klein were to be remembered for one thing, it would be for the joining of his passions for language and medicine in the production of the first translation of the Ebers Papyrus from its Ancient Egyptian hieratic script directly into English. The Ebers Papyrus remains one of our oldest and most complete contemporaneous perspectives on Ancient Egyptian healing practices, and von Klein's translation could have had a significant impact on the anglophone medical historical scholarship of the time.

The provenance of the Papyrus in many ways prefigures the biography of the man who first translated it into English. Descriptions of how it was found are rife with exaggerations and mythmaking. It is almost universally described as having been discovered between the legs of a mummy in the Valley of the Kings [26-28] before being purchased by the Prussian Egyptologist Georg Ebers in 1872. For example, von Klein attributes the papyrus's rediscovery as possessing “all the characteristics of a romance,” and relates the following tale:

A wealthy citizen of [Luxor] showed Ebers the antiquities which he, little by little, had obtained from a fellah, on the other side of the Nile. One day he exhibited one of those texts that are known under the name of “shai-en-sensen” [Book of Breathing], and a wooden Osiris statuette in which a papyrus was well concealed. […] The next day the Arab sent for Ebers and took from a tin case a well-preserved papyrus roll. According to the statement of the Egyptian possessor, the papyrus was found in a tomb in the so-called Il Assiût part of the necropolis of Thebes, between the legs of a mummy. [7]

It is not particularly surprising that Ebers's recitation of his acquisition of the Papyrus has “all the characteristics of a romance.” Ebers himself, not merely content to advance himself in the field of Egyptology, was also a novelist who wrote several *Professorenromanen* with such titles as *Uarda: A Romance of Ancient Egypt* [29], *An Egyptian Princess* [30], and *The Bride of the Nile* [31]. His popular work was said to have engendered “a short bout of Egyptomania” in the Germanophone world, leading him to become one of the most widely-read German authors at the time [32].

While both Ebers's and von Klein's accounts of the discovery of the papyrus beg credulity, its authenticity is certainly not in dispute. Its exact provenance seems to be as follows: Ebers obtained the papyrus from Edwin Smith [33, 34], the American antiquities collector for whom the oldest and most complete Ancient Egyptian surgical papyrus was named [34]. Smith purchased the medical papyrus in 1862, though he related nothing about “the natives from whom he purchased his papyrus, nor any conclusions of his own as to its origin” [26, p. 25]. In 1872, Smith sold the papyrus to Ebers for a sum that Smith had intended to be prohibitory, which forced Ebers to seek financial support to secure the purchase [33].

The value of the papyrus, however, rests not in how it was found, but in what it contains: a window into the medical epistemology of the Ancient Egyptians, which describes, as von Klein noted, “the anatomic, physiologic, pathologic and pathologico-anatomic conception of that time. Furthermore, it gives us information concerning the methods of the examination of a patient and diagnosis of the Egyptian physicians, of methods of teaching and learning, as well as of the medical standing at that time” [7, p. 1929]. Von Klein was right to characterize it as a “monument of ancient culture […] whose medical and historical value is inestimable” [7, 1928].

The manuscript that resulted from von Klein's translation was offered for pre-sale (a publisher's stipulation that in retrospect was fatal to the translation project) in an advertisement that described it as a book that would “[c]onsist of 650 pages, 7x10 inches, in two colors (red and black) similar to the original, with six plates bound in one volume” [35]. Von Klein planned to sell the translation for $5 per copy, or the equivalent of approximately $150 in 2023.

One of the men who reviewed von Klein's translation, Dr. William Fitch, the editor of *Pediatrics*, made the case for subscriptions by arguing that “[i]t is a question, in fact, whether the translation will not be lost to science […] by the author taking it with him to his bier, if he is not spared to finish the work of publication” [35]. Unfortunately for von Klein and the history of medicine, the pre-sale for the text fell short of the publisher's requisite 1000 subscriptions, only garnering approximately 600 interested readers. While von Klein ultimately offered to publish and sell the volume personally [36], the manuscript seems to have met its predicted fate, regrettably lost exactly as the author had warned.

## A MANUSCRIPT LOST, A MAN FORGOTTEN

Two central issues are raised by the sad fate of von Klein's translation. The first issue functions at a societal scale and implicates the biography of the manuscript, leading us to ask: Why was the translation not met with more interest given the “Egyptomania” of the time and its status as the first direct-to-English translation of such a significant text? The second demands a consideration of the biography of von Klein himself, the inroads he made in the anglophone history of medicine, and the dissolution of those efforts at the intimate scale of his personal life.

Substantial commentaries on von Klein's translation are found in only two sources. The first is von Klein's remarks about his project, which he made in Chicago at the Thirtieth Annual Session of the American Academy of Medicine in 1905 and published that same year in the *Journal of the American Medical Association* [7]. The second is a review of von Klein's manuscript by the physicians Bayard Holmes and P. Gad Kitterman, published in 1914 (one year after von Klein's death) by the *Lancet-Clinic Press* [37].

With so little able to be confirmed about von Klein, one may wonder whether he ever fully finished the translation and, if so, what the quality of the translation was. By the few accounts available, we are confident that the project was indeed completed. Holmes and Kitterman quote from it at length. And while those two authors were both physicians, they were also considered “student[s] of Egyptology” [38] and had published on the topic of Ancient Egyptian medicine [37]. Another reviewer, Albert Zwick, was the manager of the foreign literature section of the *Cincinnati Lancet Clinic* and likewise had an enduring, professional interest in Ancient Egypt. He even gave lectures on the “star cult” (*Sternkultus*) of that people [39] and spoke publicly about the Ebers Papyrus itself [40].

Then why did von Klein's translation fail to drum up sufficient public support for its publication? He was uniquely – and perhaps critically – situated to bring his medical knowledge (whether formalized or not) to the project. This is something that had prevented others from feeling they were adequately able to translate the papyrus. In fact, Cyril Bryan, who published an English translation of the papyrus in 1930 – seventeen years after von Klein's death – stated that, in the list of criteria for a translator of the Ancient Egyptian tome, “In the first place he must be a Physician: for a knowledge of Anatomy, Physiology, and Nosology, [is] a sine qua non” [27].

Von Klein's 1905 remarks likewise described a project that one might presume would have been of considerable interest to his audience. He characterized his work as a deliberate attempt to “cultivate the prehistoric knowledge of medicine, and to show that there existed in Ancient Egypt nearly 7,000 years ago a civilization in which medical knowledge was in a high state of cultivation” [7]. In doing so, von Klein countered the prevailing narrative (as existed both then and now) of Hippocrates as the “father” of empiric medicine, reassigning that mantel to the physicians of Ancient Egypt. He described this paradigm shift as follows:

The Anglo-Saxon nations, though renowned for deep-thinking, and philosophizing in every branch of science and art, cannot boast of a scholarship in bringing forth the first literature of the science of medicine. […] Hippocrates of Cos, who for twenty-three hundred years has been known to the world as the “Father of medicine,” and as an original observer, no longer possesses this distinction. It has been wrested from the ancient Greek by the discovery of this papyrus of a date so remote as almost to place Hippocrates within the ranks of modern physicians [7].

Although there is no record of how these remarks were received by his audience, they must have appeared radical, particularly for that time and for an audience comprised of physicians, trained as they would have been by patently Europhilic institutions. As W.E.B. Du Bois noted, Egyptology had its origins in a time when “[f]ew scholars […] dared to associate the Negro race with humanity much less with civilization” [41]. In the racist ideologies ascendant at the time, viewing Egyptians as Black meant that Egypt was unlikely to be considered the headwaters of modern medicine, even as plundered artifacts spoke to its extraordinary place in that history.

Many contemporaneous Westerners struggled to square the contributions made by a kingdom on the “Dark Continent” to the development of their own culture with these racist beliefs. Some rationalized this cognitive dissonance by drawing a red line between the “civilized” Egypt of the past and its modern Arab and African counterpart. Egyptology, the institutionally sanctioned, even lauded academic field, was solely focused on the excavation of this separate, historical society. Even the word “Egyptology” only refers to the study of the culture of antiquity, implying that Egypt “ceases to be Egypt when it ceases to be ancient” [42]. Moreover, the Egyptology of the time was a purely European (and later, Euro-American) pursuit: The façade of the Egyptian Museum in Cairo bears twenty-one names of the “founding fathers” of Egyptology, all of whom were European [42]. This is similar to von Klein's own acknowledgement of those on whose research he relied. Although he spoke with humility in his recitation of the scholars whose academic work inspired his own, it should be noted that he thanked a decidedly European cabal of Egyptologists while making no reference to the contemporary country itself.

Holmes and Kitterman's review described von Klein's translation as a considerable achievement. However, their description of the author himself suggests an additional explanation for the lack of interest in the resultant manuscript. The opening paragraph of their review placed the project outside the realm of academia, noting that “[n]othing maintains the effervescence and elasticity of mind, in young and old, like the pursuit of a hobby” [37]. The review, at turns, referred to the translational and editorial work as “recreation,” “a hobby for physicians,” and indicative of “a new fad.” Though the context of those comments does not suggest condescension, they betray the role that the politics of academia may have played in the reception of a physician's attempt at such a translation.

Von Klein was working on his translation during the latter half of the so-called Golden Age of Egyptology. Public interest in the “ancient world” was spreading across the North Atlantic in artistic, architectural, and literary manifestations, and a marketplace for the production and consumption of knowledge about the history of medicine was just beginning to take shape. The turn of the twentieth century, however, brought with it the birth of Egyptology as an official academic discipline within the United States.

The founding of the Oriental Institute (now called the Institute for the Study of Ancient Cultures) at the University of Chicago by James Henry Breasted (1865-1935), the first American to receive a doctorate in Egyptology [43], solidified the area of study as a legitimate academic domain in this country. Breasted also became the first professor of Egyptology in the United States and achieved the rank of full Professor of Egyptology and Oriental History in 1905 [43] – in the same year and in the same city in which von Klein made his remarks on the Ebers Papyrus before the American Medical Association. As noted in Breasted's 1936 memoir, this period marked a point in time at which “the era of intuitive and individualistic Egyptologists was drawing to a close and […] discipline was being introduced into the young science” [43, p. 98]. Breasted, with his continued dominance in this new field and his establishment of the University of Chicago as its American epicenter, was an architect of a transition that left von Klein and other mere “hobbyists” peripheral to the growing academic infrastructure supporting and legitimating such work.

This demarcation between the academic establishment and the “intuitive and individualistic” hobbyists is evidenced by some of the correspondence between Breasted and von Klein, or perhaps, more precisely, by its lack. On June 3, 1905, von Klein wrote to Breasted requesting a meeting “in reference to the ‘Ebers Papyrus’” which he noted he was translating [44]. The correspondence had been preceded by a May 26, 1905, letter to the Vice President of the University of Chicago, requesting a meeting between von Klein and Breasted [45]. This year was momentous for Breasted, who “took an arduous trip up the Nile” in contravention to the instructions of his physician, commenting that “I am going to Egypt, if I go on a stretcher!” [43]. Despite his scheduled travel, Breasted replied to von Klein on June 7 and agreed to meet with him “during the latter half next week” [46]. Unfortunately, no further communication, much less collaboration, appears to have materialized, and it may at least be said that von Klein was not made a part of the growing milieu of scholars in Breasted's hallowed ambit.

## THE NOBLE SAVANT AND HIS CHILDREN

While von Klein lacked the professional training and appointments that were becoming critical currency in the emerging field of American Egyptology, he was extraordinarily tenacious. This tenacity was demonstrated by his labors of translation – done over “twenty years of odd moments of a busy, useful life” [35] – and his stated intent to self-publish the manuscript, were he to fail to obtain the necessary number of subscriptions. This implicates the second question regarding the missing manuscript: Specifically, as it would have represented such a significant accomplishment in the history of medicine, why did the manuscript ultimately disappear with hardly a trace remaining? In the most cursory sense, this question has a straightforward answer. In the current authors' interviews with descendants of von Klein, the family relayed that the amateur Egyptologist requested his papers be burned upon his death and that his eldest child, Edith Zitelmann, did so.

So much for the most proximate answer. However, von Klein's life in the decade leading to his death was marked by a series of events within his immediate family that divided his attention and obligations and possibly contributed to his untimely death and to his inability to fund the alternative self-publication. Perhaps most significant was Edith's instrumental role in translating the manuscript. Edith was born in Columbus, Indiana, in 1877, far from her father's aristocratic childhood home in Prussia. She spent her early years in the rural Midwest, playing with her maternal cousins on their farms, before moving with her family to the small city of Dayton, Ohio, where she studied at a Catholic school for girls and developed her own interest in languages [48]. In 1894, von Klein took his daughter to Europe, where she studied linguistics in Rome at the Convent of the Sacred Heart [49].

**Figure 1:**
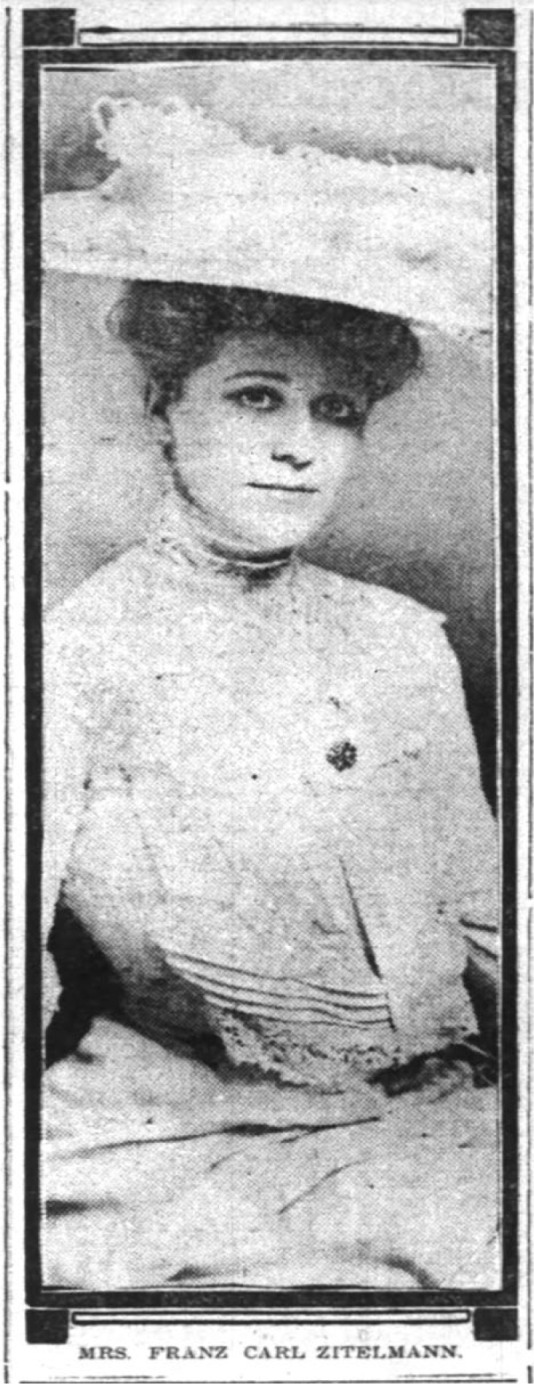
Edith née von Klein's wedding announcement [47]

Shortly after she returned from Italy, the von Kleins moved to Chicago, a city that was nearly twenty-eight times the size of Dayton and sixteen times the size of Rome. Edith quickly integrated herself in local society, joining a number of social clubs for expatriates and women interested in “foreign languages.” She joined the Società Dante Alighierei [50] and the Club Français [51], and she befriended the consulate corps from Britain, Germany, and Russia.

In commenting on Edith's participation in the translation of the Ebers Papyrus, von Klein once again adopted a rather radical view for the time, highlighting his daughter's important intellectual contributions during his concluding remarks before the American Academy of Medicine:

I also wish to express my thanks to my daughter, Edith, who for seven long years has labored by my side, inspired not merely by the devotion of filial love, but also by the same interest and purpose as that of her father, namely, to cultivate medical history and to elevate the standing of the humane and noble profession of medicine [7].

Further, as we have researched more deeply into Edith's biography, we have become increasingly confident that the translations should be regarded as the work of two scholars. Certainly, von Klein himself thought of it this way and acknowledged her in advertisements for the translation [52, 53]. Even in the announcement of Edith's engagement, her academic achievements were lauded [54].

Still, Edith seems not to have been immune to the gendered constraints placed on women at this time. Within a week of marrying German vice-consul Franz Zitelmann in July 1905, Edith left to honeymoon in Europe and then to reside indefinitely in Berlin [54]. Her descendants do not recall stories of her continuing her academic pursuits and understand her to have set such interests aside. This loss is particularly striking given that in the few historical records in which Edith made her presence known, she is remembered with praise. For example, Albert Einstein recalled meeting the Zitelmanns in Cuba in 1930, where Franz was serving as the minister of the Reich. While Einstein was dismayed at the uninteresting diplomatic receptions between which the Zitelmanns “dragged” him, he was nonetheless struck by the minister's “clever wife” [55].

Edith seems never to have spoken to her children of the Ebers Papyrus or of her efforts in translating it with her father, as such work, her descendants relayed to us, would have been considered inappropriate for the wife of a diplomat. Thus, Edith's gender almost certainly plays a role in the explanation of why the translation project – and even its memory – did not survive von Klein's death.

It was another family member, however, who seems to have created ultimately insurmountable challenges for von Klein's work. In 1913, his oldest son, Edmund, was arrested at the iconic Blackstone Hotel in Chicago, on charges of polygamy and larceny. Edmund had entered a second marriage under an assumed name and subsequently stolen some $3,300 worth of jewelry from his new wife (the equivalent of nearly $100,000 in 2023). His father drained himself of energy and financial resources to help his son, reportedly telling him, “I do not believe you have gone wrong and will aid you” [56]. Nonetheless, Edmund was eventually indicted one day after his father's death, on December 13, 1913 [57].

In many announcements of Carl von Klein's passing in 1913, the authors noted the corrosive effects of Edmund's behaviors on the elder von Klein's well-being. The translator, now a gray-haired seventy-one-year-old, was said to have collapsed in tears upon learning of the accusations made against his son [59]. He publicly recommitted himself to supporting the troubled Edmund through his legal ordeal, but despite his obdurate optimism, the senior von Klein's health quickly deteriorated. Moreover, he depleted his remaining financial resources, paying for Edmund's exorbitant bails and helping to fund his multiple attorneys. Even the *New York Times* linked the arrest of Edmund to the physical exhaustion of the researcher of the “medical lore of the ancients” [60]. With his health and money spent, von Klein died on December 12, 1913.

**Figure 2:**
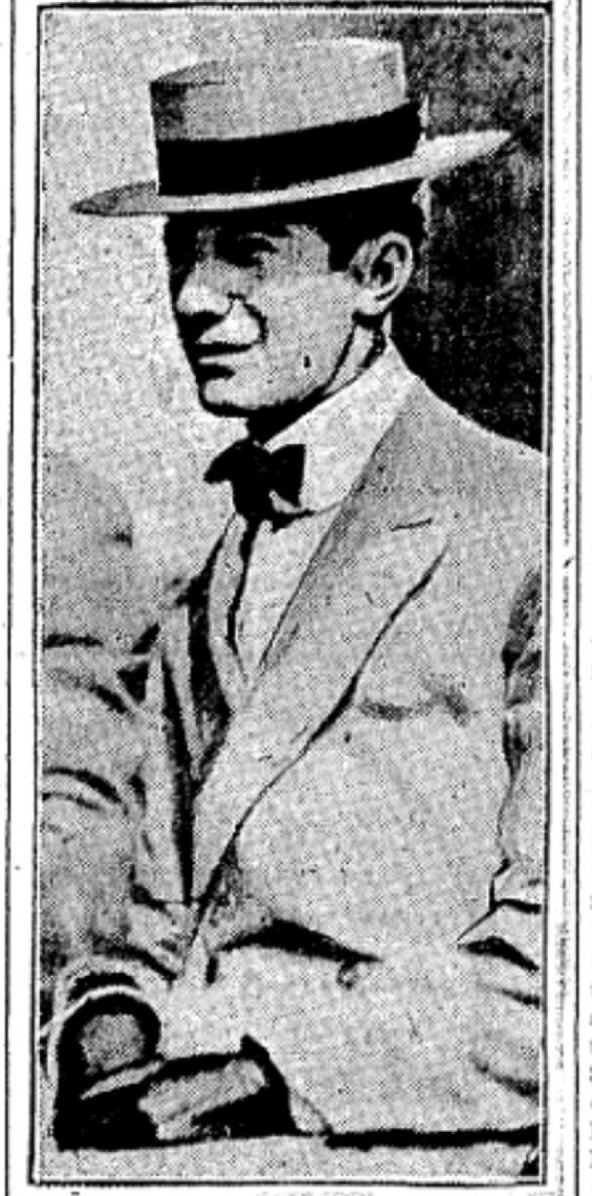
Edmund von Klein [58]

## CONCLUSION

Together with his daughter Edith Zitelmann, Dr. Carl von Klein created the first direct-to-English translation of the Ebers Papyrus, an artifact that played a critical role in disrupting the then-dominant narrative of the history of medicine. The father-and-daughter team participated in paradigm-shifting work that relocated medicine's origins from the Greek island of Cos to the banks of the Nile. That the only record of this extraordinary effort is found in brief reviews and stray comments in the works of von Klein's colleagues is a testament to the finitude and fragility of human knowledge. This tenacious immigrant surgeon was hindered by both societal and personal factors beyond his control – the pretentions of the burgeoning academic field of Egyptology; the departure of Edith, his collaborator; and the draining influence of his wayward son, Edmund. An English-language version of the Ebers Papyrus would not become available until nearly two decades after von Klein's death [27], potentially exacting a significant delay in the development of the history of medicine in the Anglophone world. While his original translation indeed appears to be permanently lost, the current authors propose to ameliorate this disappearance by explicating the extrinsic forces that have threatened such projects of knowledge-making both at the turn of the 1900s and that continue to this day.
